# Ultra-Low Percolation Threshold Induced by Thermal Treatments in Co-Continuous Blend-Based PP/PS/MWCNTs Nanocomposites

**DOI:** 10.3390/nano11061620

**Published:** 2021-06-21

**Authors:** Daria Strugova, José Carlos Ferreira Junior, Éric David, Nicole R. Demarquette

**Affiliations:** Mechanical Engineering Department, École de Technologie Supérieure, Montréal, QC H3C 1K3, Canada; daria.strugova.1@ens.etsmtl.ca (D.S.); jose-carlos.ferreira-junior.1@ens.etsmtl.ca (J.C.F.J.); Eric.David@etsmtl.ca (É.D.)

**Keywords:** electrical percolation threshold, electrical conductivity, thermoplastic composites, polymer blend, multi-wall carbon nanotubes, polymer crystallization

## Abstract

The effect of the crystallization of polypropylene (PP) forming an immiscible polymer blend with polystyrene (PS) containing conductive multi-wall carbon nanotubes (MWCNTs) on its electrical conductivity and electrical percolation threshold (PT) was investigated in this work. PP/PS/MWCNTs composites with a co-continuous morphology and a concentration of MWCNTs ranging from 0 to 2 wt.% were obtained. The PT was greatly reduced by a two-step approach. First, a 50% reduction in the PT was achieved by using the effect of double percolation in the blend system compared to PP/MWCNTs. Second, with the additional thermal treatments, referred to as slow-cooling treatment (with the cooling rate 0.5 °C/min), and isothermal treatment (at 135 °C for 15 min), ultra-low PT values were achieved for the PP/PS/MWCNTs system. A 0.06 wt.% of MWCNTs was attained upon the use of the slow-cooling treatment and 0.08 wt.% of MWCNTs upon the isothermal treatment. This reduction is attributed to PP crystals’ volume exclusion, with no alteration in the blend morphology.

## 1. Introduction

Conductive thermoplastic composites have gained a lot of attention in different research fields since they are used in a variety of industrial applications. Among their many applications, these materials are used in sensors, intelligent medical devices, energy harvesting, actuators, flexible electronics, robotics, static dissipation and electromagnetic interference (EMI) shielding. The interest in these composite materials stems from their advantages, namely the ability to achieve the electrical conductivity in a wide range, processability into products of complex shapes, as well as flexibility, lightweight, and corrosion resistance [1,2,3,4,5,6,7,8,9,10,11,12].

Conductive nanocomposites are obtained by dispersing electrically conductive nanoparticles (particles with at least one of their dimensions ranging from 1 to 100 nm) within a matrix. The most used nanoparticles to obtain electrically conductive polymers are carbon black (CB) [13,14,15,16,17,18,19,20,21,22,23], carbon nanotubes (CNTs) [2,3,4,5,6,7,8,24,25,26,27,28], and graphene [12,29,30,31,32,33,34,35,36,37]. Due to the advantages conductive thermoplastic composites present, significant research has been conducted towards achieving certain desired properties at low filler concentrations, primarily by reducing the percolation threshold (PT). The PT is defined as the concentration of particles at which the composite starts to be conductive.

Several methods to reduce the PT concentration have been reported in the literature. One of the simplest methods is to use well-dispersed conductive particles with the highest possible aspect ratio as a filler. This has been experimentally found to lower the PT as was predicted by Bruggeman, V.D., and Böttcher, C. in the 1940s [38,39]. In order to further reduce the PT, more recent studies have dealt with the modification of nanoparticles, whilst others have made use of the controlled matrix morphology to tailor the location of nanoparticles. In particular, the use of immiscible polymer blends (PBs), presenting a co-continuous morphology, has been suggested. By controlling the interfacial tension between the filler and the polymers forming the blend, one can tailor the location of the conductive filler at the interface, thus lowering the PT. Depending on the used binary blend, the nanoparticles were added alone or with a compatibilizer [6,9,24,25,26,40,41,42,43]. For example, Chen, J. et al. have shown that introducing 5 wt.% of SEBSg-MA in PP/PS (70/30 wt.%) co-continuous matrix reduces PT from 1.22 wt.% for PP/PS/MWCNTs to 0.66 wt.% for PP+SEBS-g-MA/PS/MWCNTs [44]. Other additives like graphene, graphene oxide (GO), organoclay, CB, noncovalent and covalent modifiers were also reported in the literature to reduce the PT of PB/CNTs co-continuous systems [9,11,24,28,41,45,46,47].

Furthermore, other researchers suggested that the thermal annealing of polymer/filler or PB/filler composites above the melting or softening temperature could further decrease the PT and improve the electrical conductivity of a system [11,14,18,29,30,31,35,42,48,49,50,51,52]. In polymer blend-based composites, this effect is usually accompanied by changes in the blend morphology. First, the morphology coarsens (the domain size becomes larger) and then it is stabilized by increasing the annealing time of the filled composites. This leads to the easier creation of the conductive filler network due to the reduction in the interphase area where the filler is distributed. One of the most dramatic effects was, for example, achieved by Chen, Y. et al. who showed that annealing at 200 °C for 2 h decreased PT from 0.48 to 0.09 wt.% for a PP/PMMA (30/70 wt.%) co-continuous blend containing MWNTs [49]. All these strategies have been used for polymer pairs containing both amorphous and semi-crystalline polymers. However, the influence of crystallization on PT and electrical conductivity for immiscible polymer blends, containing at least one semi-crystalline polymer, has to date not been adequately evaluated. 

To our knowledge, there are few studies that have evaluated the effect of crystallization on the PT of polymer/filler conductive composites [53,54,55]. Wang, J. et al. have investigated the effect of the cooling rate on the electrical conductivity of PP/MWCNTs composites containing a sorbitol-based external nucleating agent (NA). They showed that the PT was reduced for both PP/MWCNTs and PP/MWCNTs/NA from 0.75 wt.% at a fast-cooling rate of 150 °C/min to 0.36 wt.% at a slow-cooling rate of 1.5 °C/min [53]. Huang, C. et al. have shown the effect of matrix crystallinity on the PT of PLLA/MWCNTs composites containing 0.15 wt.% of a NA. The PT was reduced from 0.96 to 0.75 wt.% for the samples which were treated for 0.1 and 6 min, respectively, at 130 °C [55]. Other researchers have reported the effect of stereocomplex (SC) crystallization on the PT and electrical conductivity of miscible PLLA/PDLA blend composites, containing a conductive filler [56,57,58,59,60]. In these cases, the change in PT happens due to the volume exclusion effect of stereocomplex crystals. 

The present study aimed to shed light on the effect of the semi-crystalline polymer’s crystallization within the PB on the reduction in the percolation threshold. In particular, for this we chose PP and PP/PS blend-based composites containing MWCNTs obtained by the melt-mixing process. It will be shown that two types of treatments aiming to affect PP crystal growth can significantly improve the electrical conductivity and reduce the PT to ultra-low values. These treatments are proposed to achieve lower PTs for other semi-crystalline-based PB co-continuous systems. In addition, it will be shown that the proposed treatments do not significantly change the PB morphology compared to thermal annealing above the melting or softening temperature.

## 2. Materials and Methods

### 2.1. Materials

Commercial polypropylene (PP)—PP4712E1 grade from Exxon Mobile with a density 0.9 g·cm^−3^—and polystyrene (PS)—MC3650 from PolyOne with a density 1.04 g·cm^−3^—were used in this work. Multi-walled carbon nanotubes (MWCNTs) grade NC7000^TM^ from Nanocyl with an average diameter and a length of 9.5 nm and 1.5 μm, respectively (aspect ratio ~ 160), and with a nominal electrical conductivity of 10^6^ S·m^−1^, were used as the conductive filler in the composites.

### 2.2. Processing

#### 2.2.1. Extrusion

The materials were prepared by a melt-mixing process using a Haake Rheomix OS PTW16 twin-screw extruder (Thermo Fisher Scientific Inc., Waltham, MA, USA). The temperature was fixed at 220 °C in all zones, and the screw speed was adjusted to 100 rpm for all compositions. First, a masterbatch of PP with 10 wt.% of MWCNTs was prepared. Second, PP composites with a MWCNTs concentration varying from 0 to 2 wt.%, as well as co-continuous morphology 50/50 PP/PS blends with MWCNTs concentration, also varying from 0 to 2 wt.%, were prepared by dilution of the PP/MWCNTs masterbatch.

To achieve a co-continuous morphology for PP/PS/MWCNTs composites, the calculation of a co-continuous range based on viscosity measurements both PP and PS should be done. The viscosity of both PP and PS was measured using a capillary rheometer at a temperature of 200 °C. For the effective shear rate of 100 s^−1^ experienced in the twin-screw extruder, both polymers manifested similar viscosities (as can be seen in Figure S1 in the Supplementary Materials) [61,62]. A 50/50 wt.% PP/PS concentration was, therefore, chosen following the analysis of Jordhamo, G. et al. [63].

#### 2.2.2. Thermal Treatments

**Figure 1:**
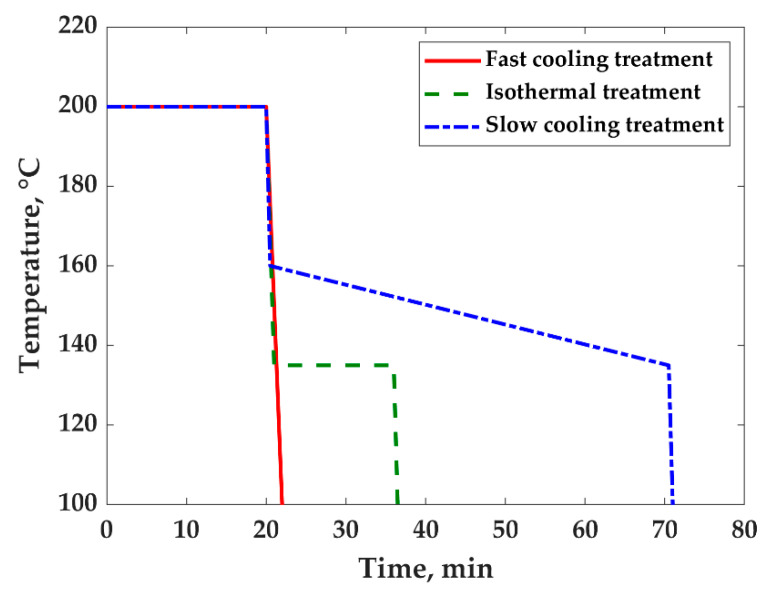
Temperature vs. time for fast-cooling treatment, isothermal treatment, and slow-cooling treatment.

Three thermal treatments were performed by compression molding and were designated here as: (1) fast cooling; (2) slow cooling; and (3) isothermal. Figure 1 shows a schematic of the temperature vs. time profile for all thermal treatments.

The three treatments consisted of three steps each, with the first two steps being the same and a third one differing for each treatment. The first step was at a temperature of 200 °C under constant pressure of 0.8 MPa for 10 min. The second one was at a temperature of 200 °C under a pressure of 10 MPa for an additional 10 min.

1. For the fast-cooling treatment, the third step was fast cooling to room temperature, which was performed at a rate of 50 °C/min under a pressure of 10 MPa. The whole treatment, all three steps, took 22 min (Figure 1);
2. For the slow-cooling treatment, the third step was fast cooling to 160 °C and a slow cooling from 160 to 135°C at a rate of 0.5 °C/min, followed by fast cooling from 135 °C to room temperature under a pressure of 10 MPa. The whole treatment took 1 h 10 min (Figure 1). The starting temperature of the treatment (160 °C) was chosen to prevent the so-called annealing effect [11,14,29,30,31,49] which could involve the coarsening of blend morphology. The temperature of 135 °C at the end of the treatment corresponds to the highest onset temperature of crystallization, evaluated by the DSC analysis.
3. For the isothermal treatment, the third step was fast cooling to 135 °C which was maintained for 15 min. Then, the sample was fast cooled to room temperature under a pressure of 10 MPa. The whole treatment took 36 min (Figure 1).

Fast- and slow-cooling treatments were carried out to study how the cooling rate, which has a direct effect on the crystallization kinetics and crystals morphology, affects electrical conductivity due to the volume exclusion effect. Slow cooling was performed at 0.5 °C/min to allow the growth of larger crystals.

### 2.3. Characterization

#### 2.3.1. Crystallization Studies

Differential scanning calorimetry (DSC) was performed using a Pyris 1 Differential Scanning Calorimeter (PerkinElmer, Waltham, MA, USA). The nitrogen gas flow rate was set to 20 mL/min. The samples were encapsulated in standard aluminum pans and covers. The DSC was calibrated using indium and zinc standards. 

Two different cycles were used to determine the crystallinity of the composites as well as the crystallization kinetics.

- Non-Isothermal Crystallization

The samples were heated from 50 to 200 °C at 10 °C/min and then cooled down from 200 to 50 °C at 10 °C/min under nitrogen atmosphere. This thermal cycle was performed twice for all samples to erase the thermo-mechanical history of the samples. The data from the second heating and cooling cycle were used for calculations. Prior to that, the same thermal cycle was run with empty pans for getting a baseline.

The crystallinity for all compositions was calculated using Equation (1):

(1) $$X_{c}=\frac{\Delta H}{\left(1-w\right)\Delta H_{m}},$$

where $X_{c}$ is the weight fraction of the crystalline phase, ΔH is the heat of fusion of the sample, $\Delta H_{m}$ is the heat of fusion of 100% crystalline PP (207 J/g) [64], and w is the MWCNTs’ weight fraction for PP/MWCNTs composites and (MWCNTs + PS) weight fraction for PP/PS/MWCNTs composites.

- Isothermal Crystallization

The samples were heated from 50 to 200 °C at 50 °C/min and kept at this temperature for 5 min to eliminate any previous thermal history; then, they were cooled to 135 °C at 50 °C/min and kept at 135 °C for 30 min for isothermal crystallization. Again, in this case, prior to testing the sample, the same thermal cycle was run with empty pans to obtain a baseline.

#### 2.3.2. Polarized Optical Microscopy

The crystals’ growth and their morphology were studied using polarized optical microscopy (POM)—OLYMPUS BX51 microscope (Olympus Co., Tokyo, Japan) equipped with a hot stage. The morphology evolution (crystal growth) at 135 °C was observed. In this case, the heat treatment was performed without any applied pressure. Furthermore, the morphology of the crystals of pure PP and PP/MWCNTs composites at room temperature obtained by fast-cooling, isothermal, and slow-cooling treatments as described above was also observed.

#### 2.3.3. Electrical Conductivity

The electrical properties as a function of frequency for all compositions were evaluated using a broadband dielectric spectrometer (BDS) (Novocontrol Technologies GmbH & Co. KG, Montabaur, Germany) in the frequency range from 10^−2^ to 3 × 10^5^ Hz under an excitation voltage of 3 VRMS applied across the sample.

The electrical conductivity was evaluated from the measurement of the AC complex conductivity as a function of frequency—$\sigma^{*}\left(\omega \right)$, which is related to the complex permittivity by

(2) $$\sigma^{*}\left(\omega \right)=j\omega \varepsilon_{0}\varepsilon^{*}\left(\omega \right),$$

where ω is the frequency, $\varepsilon_{0}$ is the vacuum permittivity and $\varepsilon^{*}\left(\omega \right)$ is the complex permittivity which includes the contributions of the electrical conductivity and can be expressed as

(3) $$\varepsilon^{*}\left(\omega \right)=\varepsilon^{\prime }\left(\omega \right)-j\varepsilon_{tot}^{\prime \prime }\left(\omega \right)=\varepsilon^{\prime }\left(\omega \right)-j\left(\varepsilon_{P}^{\prime \prime }\left(\omega \right)+\frac{\sigma }{\omega \varepsilon_{0}}\right),$$

where $\varepsilon^{\prime }$ is the real part of the complex permittivity, $\varepsilon_{tot}^{\prime \prime }\left(\omega \right)$ the total imaginary part, $\varepsilon_{P}^{\prime \prime }$ represents the imaginary part of the permittivity due to the polarization phenomena, and σ is an electrical conductivity. By combining these two equations, we can obtain:

(4) $$\sigma^{*}\left(\omega \right)=j\omega \varepsilon_{0}\varepsilon^{*}\left(\omega \right)=j\omega \varepsilon_{0}\left(\varepsilon^{\prime }\left(\omega \right)-j\left(\varepsilon_{P}^{\prime \prime }\left(\omega \right)+\frac{\sigma }{\omega \varepsilon_{0}}\right)\right)=\sigma +\omega \varepsilon_{0}\varepsilon_{P}^{\prime \prime }\left(\omega \right)+j\omega \varepsilon_{0}\varepsilon^{\prime }\left(\omega \right),$$

where the real part of the complex conductivity is:

(5) $$\sigma^{\prime }\left(\omega \right)=\sigma +\omega \varepsilon_{0}\varepsilon_{P}^{\prime \prime }\left(\omega \right),$$

The equipment measures the total imaginary part—$\varepsilon_{tot}^{\prime \prime }\left(\omega \right)$ since the device cannot distinguish between the two contributions. Accordingly, the electrical conductivity cannot be formally isolated. However, since it does not increase with frequency unlike the contribution from $\varepsilon_{P}^{\prime \prime }\left(\omega \right)$, the occurrence of a low frequency plateau in the plot of $\sigma^{\prime }\left(\omega \right)$ as a function of frequency indicates that the value of the electrical conductivity dominates the real part of the complex conductivity that then becomes very close to the true DC conductivity at low frequencies. The electrical conductivity values presented in this work refer to the value of $\sigma^{\prime }\left(\omega \right)$ at the lowest frequency (1 × 10^−2^ Hz). Consequently, this value is always higher than the true conductivity, particularly below the percolation threshold, however, once the percolation threshold is reached, it gives a very good approximation of the conductivity. Disks of 25 mm in diameter and 1 mm in thickness, covered on both sides with 20 nm of gold, were used for the measurements.

#### 2.3.4. Scanning Electron Microscopy

The morphology of PP/PS/MWCNTs composites for all treatments was observed by scanning electron microscopy (SEM) using a S3600 Hitachi microscope (Hitachi, Ltd., Tokyo, Japan) in the secondary electrons mode. The samples were fractured in liquid nitrogen and then polystyrene phase was extracted by using butanone at room temperature, under continuous stirring for two hours. Then, the samples were dried under vacuum at room temperature during 12 h. After drying, the samples were covered with gold by using a gold sputter coater, model K550X. All porous samples after PS extraction were imaged at an accelerating voltage of 5 kV.

## 3. Results

### 3.1. Effect of the Treatments on Electrical Conductivity and Morphology of PP/PS/MWCNTs Composites

**Figure 2:**
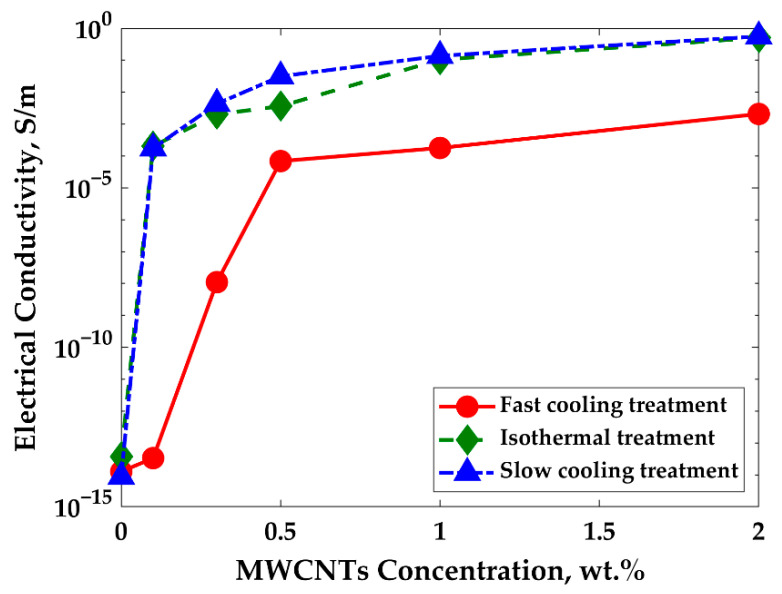
Effect of treatments on the electrical conductivity of PP/PS/MWCNTs composites as a function of MWCNTs concentration.

Figure 2 shows the electrical conductivity as a function of MWCNTs mass fraction for PP/PS/MWCNTs composites after fast-cooling, isothermal, and slow-cooling treatments. Equation (6) was used to calculate the percolation threshold of composites that underwent the different thermal treatments:

(6) $$\sigma =k\cdot {\left(p-p_{c}\right)}^{t},\;\mathrm{with}\;p>p_{c},$$

where σ is the electrical conductivity of the composite, p is the mass fraction of MWCNTs, $p_{c}$ is the percolation threshold (PT), t is a fitted exponent that depends, only, on the dimensionality of the system, and k is a scaling factor. It should be noted that this equation is valid for $p>p_{c}$.

A linear regression fit was employed to determine the percolation threshold, log(σ) vs. $\log\left(p-p_{c}\right)$. The results of these fits for each treatment of PP/PS/MWCNTs composites are presented in Table 1.

The results presented in Figure 2 and Table 1 show that the isothermal and slow-cooling treatments resulted in a much lower percolation threshold. The percolation threshold was drastically reduced from 0.28 wt.% to 0.08 wt.% and 0.06 wt.% of MWCNTs for the isothermal treatment and slow-cooling treatment, respectively, where an increase of 10 orders of magnitude in electrical conductivity was observed. Here, a double effect on reducing the PT of PP/PS/MWCNTs composites was achieved. On the one hand, the PT was reduced due to the effect of the double percolation using the co-continuous morphology of PP/PS/MWCNTs composites. Indeed, the PT was reduced from 0.6 wt.% for PP/MWCNTs composites (as can be seen in Figure S2 and Table S1 in the Supplementary Materials) to 0.28 wt.% for PP/PS/MWCNTs composites for the fast-cooling treatment. On the other hand, the ultra-low PT was achieved for PP/PS/MWCNTs composites after the isothermal and slow-cooling treatments. These results can be explained by the exclusion of the MWCNTs by the PP crystalline structure, as was observed by Wang, J. et al. [53]. 

PP/PS/MWCNTs composites formed a co-continuous structure, where the quantification of morphology can be done by calculating the characteristic domain size ξ—total area of the SEM image per total interfacial length between PP and PS phase. The evolution of the morphology of the blends for all composites for different treatments was investigated using SEM images. The characteristic domain size—ξ was studied by averaging at least five different SEM images for the same treatment and the same concentration of MWCNTs by using the following equation [30,31]:

(7) $$\xi =\frac{A_{SEM}}{L_{int}},$$

where $A_{SEM}$ is the total area of the SEM image and $L_{int}$ is the interface length between two phases estimated using a homemade image analysis script (as can be seen in Figure S3 in Supplementary Materials, which shows how $L_{int}$ was estimated).

**Figure 3:**
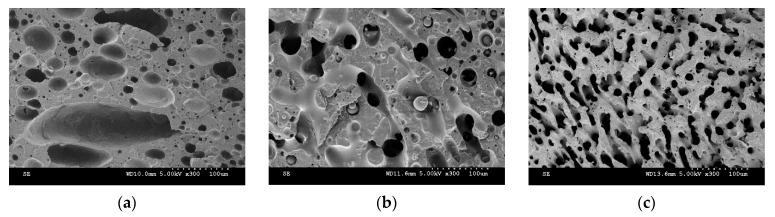
Morphology evolution of selected PP/PS/MWCNTs composites after fast-cooling treatment for: (**a**) 0 wt.% of MWCNTs; (**b**) 0.1 wt.% of MWCNTs; and (**c**) 0.5 wt.% of MWCNTs.

**Figure 4:**
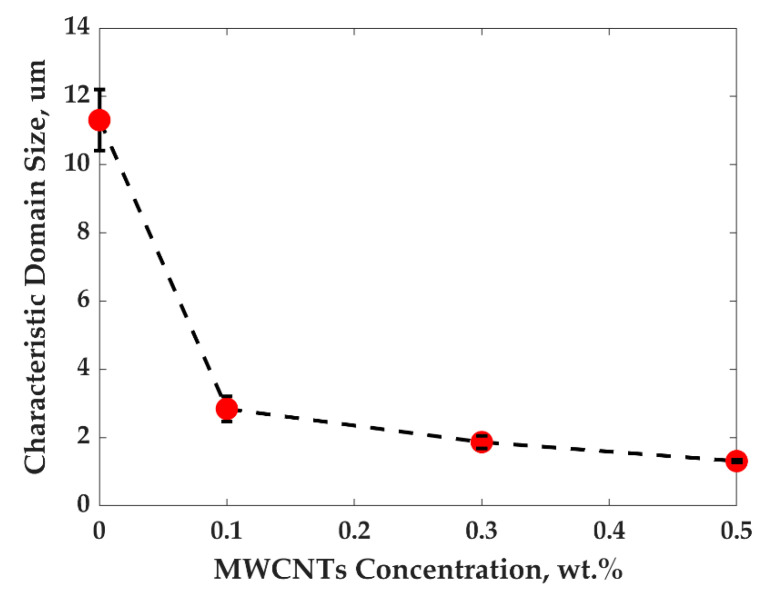
Characteristic domain size of PP/PS/MWCNTs composites after fast cooling as a function of MWCNTs concentration.

Figure 3 and Figure 4 show SEM images and the plot of characteristic domain size of PP/PS/MWCNTs composites for which the PS phase was extracted with different filler concentrations after the fast-cooling treatment. It can be seen that upon the addition of MWCNTs the characteristic domain size drastically decreases from 11.3 μm to 1.3 μm for neat PP/PS blend and PP/PS/MWCNTs composite with 0.5 wt.% of MWCNTs, respectively. Indeed, the composites were prepared by adding PP/MWCNTs to PS. PP/MWCNTs presents a higher viscosity than pure PP and transfers, therefore, more stress to the PS phase, and resulting in a finer morphology. Furthermore, the better affinity of MWCNTs to PS favors its migration to the PS phase, preventing its coalescence and coarsening, which is leading to drastic decrease for the characteristic domain size [29,30]. This decrease indicates that MWCNTs refined the morphology and that the number of viable electrical paths is increased, explaining the increase in electrical conductivity shown Figure 2.

**Figure 5:**
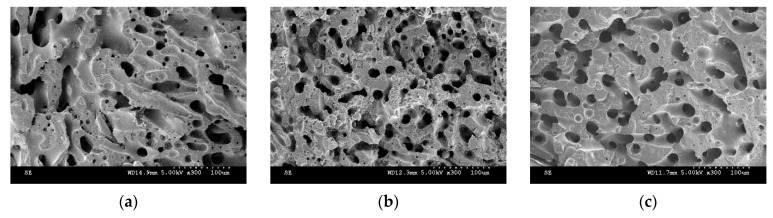
Morphology of the selected PP/PS/MWCNTs composites with 0.3 wt.% of MWCNTs for (**a**) fast-cooling treatment; (**b**) isothermal treatment; and (**c**) slow-cooling treatment.

**Figure 6:**
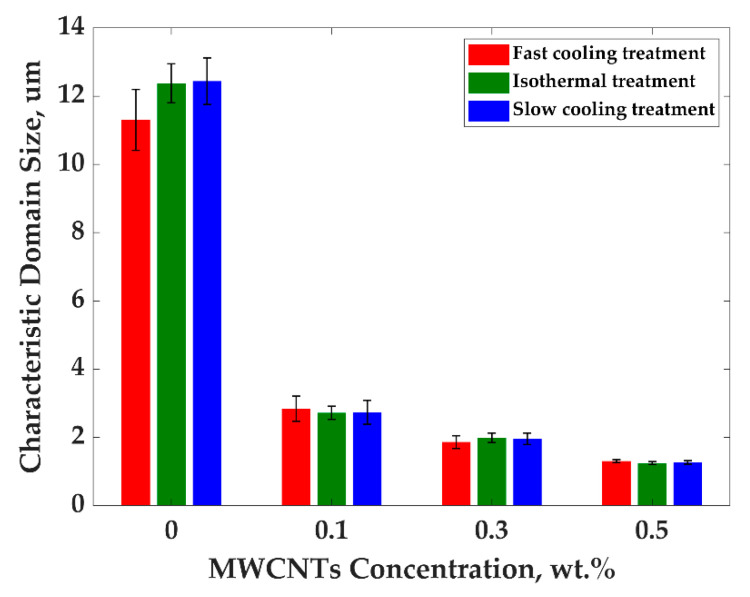
Characteristic domain size of ζ vs. MWCNTs concentration for different treatments for PP/PS/MWCNTs composites.

The SEM micrographs for PP/PS/MWCNTs composites with 0.3 wt.% of MWCNTs for each treatment are reported in Figure 5a–c. Figure 6 shows the characteristic domain size (ξ) of the PP/PS blends as a function of MWCNTs concentration for different treatments. It can be seen that, for the composites with the same amount of MWCNTs—but for different treatments—the morphology of the blends did not change, although a larger electrical conductivity was observed upon the slow-cooling and isothermal treatments. For example, the characteristic domain size is 2.8 μm, 2.7 μm, and 2.7 μm for the PP/PS/MWCNTs composite with 0.1 wt.% of MWCNTs subjected to fast-cooling, isothermal, and slow-cooling treatments, respectively. However, the electrical conductivity is 3.4 × 10^−14^ S/m, 2.0 × 10^−4^ S/m, and 1.7 × 10^−4^ S/m for the PP/PS/MWCNTs composite with 0.1 wt.% of MWCNTs subjected to fast-cooling, isothermal, and slow-cooling treatments, respectively. These observations indicate that the increase in electrical conductivity upon thermal treatments, in the case of the blends studied here, may not originate from an evolution of the blend morphology as suggested by several researchers [11,14,18,29,30,31,42,48,50]. Rather, this stems from an evolution of the crystalline morphology of the semi-crystalline polymer. The next section will present an analysis of the crystallization of PP that will help to understand the obtained results.

### 3.2. Crystallization Behavior and Electrical Conductivity of PP/MWCNTs and PP/PS/MWCNTs Nanocomposites

DSC thermograms of non-isothermal and isothermal crystallization were used to investigate the crystallization state as well as the isothermal crystallization behavior of PP/MWCNTs and PP/PS/MWCNTs nanocomposites.

**Figure 7:**
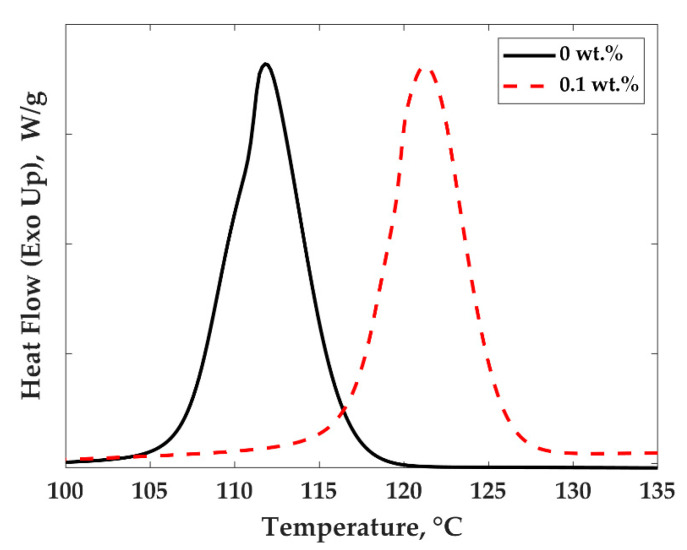
Typical non-isothermal crystallization of PP and PP/MWCNTs curves with different MWCNTs wt.%.

**Figure 8:**
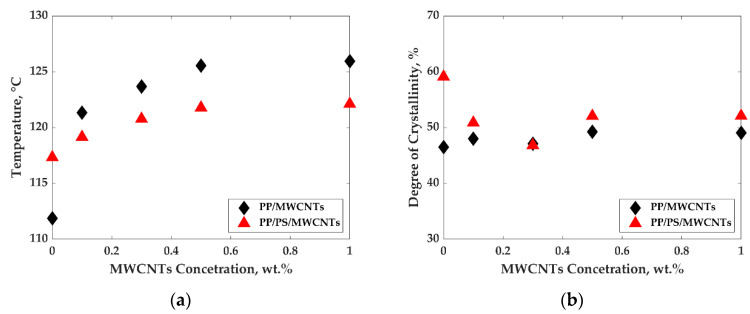
Effect of MWCNTs on non-isothermal crystallization of PP for both PP/MWCNTs and PP/PS/MWCNTs composites: (**a**) influence of MWCNTs concentration on crystallization temperature (peak); and (**b**) the effect of MWCNTs amount on degree of crystallinity during non-isothermal cooling from 200 °C to 50 °C at 10 °C/min.

Figure 7 shows typical thermograms obtained by DSC during cooling scans at 10 °C/min for PP and the PP/MWCNTs composite containing 0.1 wt.% of MWCNTs. Similar results were obtained for all composites. These curves were used to infer the peak crystallization temperature, the onset, and end of crystallization, as well as the crystallinity rate for all composites. Melting temperatures for all compositions were also determined by DSC during heating scans at 10 °C/min. The data for peak crystallization temperature (Tc), as well as the degree of crystallinity as a function of MWCNTs concentration for both PP/MWCNTs and PP/PS/MWCNTs, are presented in Figure 8a,b. The data for melting temperatures, the onset, and end of crystallization temperatures can be found in Table S2 in the Supplementary Materials.

Figure 8a,b present the crystallization behavior of PP/MWCNTs and PP/PS/MWCNTs nanocomposites. The peak crystallization temperature (Tc) increases from 112 °C to 126 °C for PP/MWCNTs composites and from 117 to 122 °C for PP/PS/MWCNTs composites as the MWCNTs concentration is increased from 0 to 1 wt.% (Figure 8a). This increase in crystallization temperature originates from the nucleating effect of the MWCNTs. The effect is more pronounced for PP/MWCNTs composites as opposed to that of PP/PS/MWCNTs composites. This could be due to a favored location of the MWCNTs at the interface between both phases of the blends. 

Finally, the addition of MWCNTs and the blending of polymers do not change the degree of crystallinity of PP in the case of PP/MWCNTs composites, as well as in the case of PP/PS/MWCNTs composites (Figure 8b).

**Figure 9:**
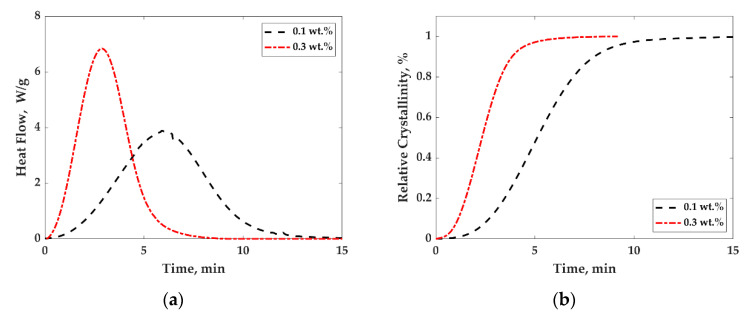
(**a**) Heat flow as a function of time for PP/MWCNTs composites with different MWCNTs wt.% at 135 °C; (**b**) relative crystallinity of PP/MWCNTs composites with different MWCNTs wt.% at 135 °C.

Figure 9a,b show typical heat flow curves and the relative crystallinity curves as a function of time during isothermal crystallization tests at a temperature of 135 °C, for the composites studied here to which MWCNTs were added. Similar curves were obtained for all composites. The heat flow curves (Figure 9a) were used to infer relative crystallinity—X(t) as a function of time, which can be obtained from the area under the exothermic peak up to time t, divided by the total exothermic peak area as expressed in Equation (8) [65]:

(8) $$X\left(t\right)=\frac{\int_{0}^{t}\frac{dH_{c}}{dt}\times dt}{\int_{0}^{\infty }\frac{dH_{c}}{dt}\times dt},$$

$dH_{c}$ is the heat flow required for crystallization for a certain time dt.

**Figure 10:**
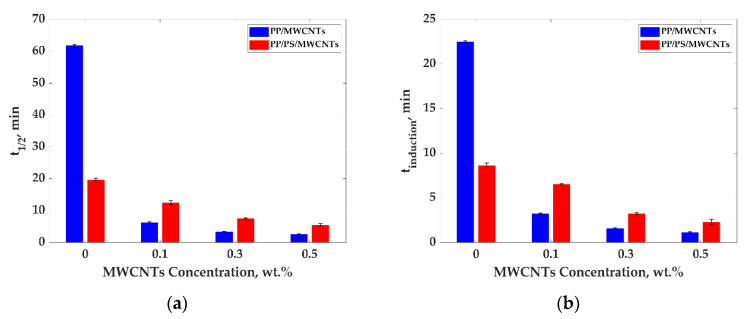
(**a**) Crystallization half time and (**b**) induction time for PP/MWCNTs and PP/PS/MWCNTs composites at 135 °C.

Figure 9b shows the relative crystallinity curves as a function of time (obtained using Equation (8) from Figure 9a data) for the composites studied here at different MWCNTs concentrations and at 135 °C. Similar curves were obtained for all composites and were used to infer the crystallization half time (t_1/2_) which is the time required to complete 50% of the crystallization and the induction time which is the time at which the nuclei start to form. The data for t_1/2_ and induction time as a function of MWCNTs concentration are shown in Figure 10a,b.

Figure 10a,b present crystallization half time (a) and induction time (b) for PP/MWCNTs and PP/PS/MWCNTs composites with different MWCNTs concentrations at 135 °C.

The results presented in Figure 10a show that upon the addition of MWCNTs for PP/MWCNTs composites, the crystallization half time drastically decreases, indicating that MWCNTs act as a nucleating agent. The crystallization half time was found to be equal to 62, 6, 3, and 2.6 min for composites with 0, 0.1, 0.3, and 0.5 wt.%, respectively. For PP/PS/MWCNTs composites, not only the effect of MWCNTs amount on relative crystallinity should be taken into account, but also the matrix composition. The crystallization half time did not significantly change with the increase in the MWCNTs concentration. The crystallization half time was determined to be 12, 7, and 5 min for composites with 0.1, 0.3, and 0.5 wt.%, respectively, which is approximately two times more than that for PP/MWCNTs composites, with the same MWCNTs amount. This could be caused by the selective localization of MWCNTs at the interface inside the PP/PS matrix, which weakens the nucleation effect since nucleation is not a factor for PS. However, t_1/2_ for the pure PP/PS blend, it is three times smaller than for pure PP. The presence of PS in the mixture could have changed the energy needed for the ultimate crystallization of PP. The same behaviour was observed for the induction time of crystallization for both PP/MWCNTs and PP/PS/MWCNTs (Figure 10b).

**Figure 11:**
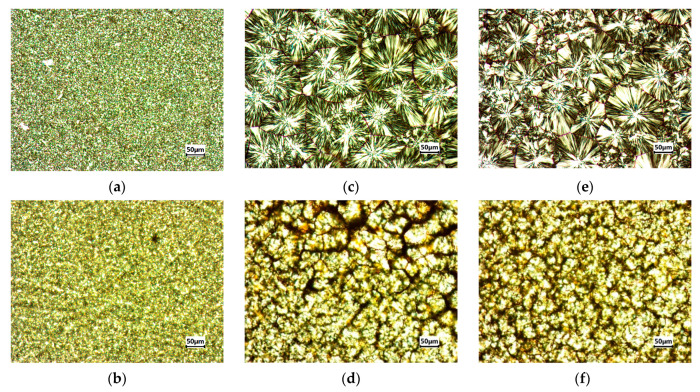
Microscopic observations of PP pure and PP/MWCNTs with 0.1 wt.% of MWCNTs for fast-cooling treatment (**a**,**b**); isothermal treatment (**c**,**d**); and slow-cooling treatment (**e**,**f**).

The crystallization of pure PP and PP/MWCNTs with 0.1 wt.% of MWCNTs after the fast-cooling, isothermal, and slow-cooling treatments was investigated by polarized optical microscopy, and the crystal morphology is shown in Figure 11a–f. A concentration of 0.1 wt.% was chosen as it is not possible to visualize by optical microscopy, the spherulites for higher concentrations of MWCNTs. The treatment temperature of 135 °C was chosen for isothermal and slow-cooling treatment to obtain larger crystals as mentioned previously. It can be clearly seen that the cooling rate affects the crystal size when fast cooling is compared to the other two treatments. The crystals of pure PP—which was fast cooled—are so small that they cannot be easily identified compared to the crystals obtained from isothermal and slow-cooling treatments, where the size is around 100 μm for both treatments. The crystals size for the PP/MWCNTs composite with 0.1 wt.% of MWCNTs decreased compared to neat PP. Nevertheless, there is a big difference in the crystal size for the samples, which were treated by fast-cooling treatment compared to isothermal and slow-cooling treatments, although crystal sizes for composites are difficult to identify by POM. 

**Figure 12:**
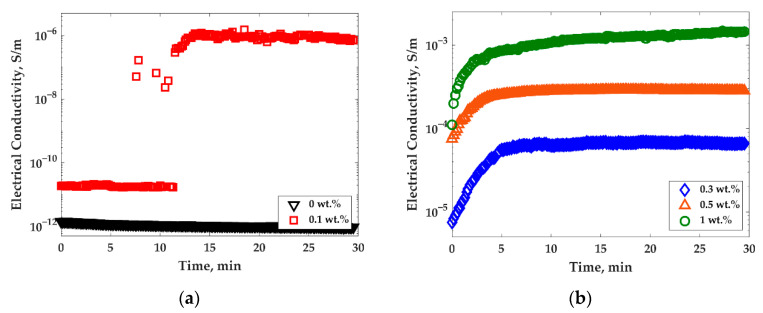
Electrical conductivity as a function of time for PP/PS/MWCNTs composite with different concentrations of MWCNTs measured every 10 s at 1 Hz of frequency and 135 °C for: (**a**) 0–0.1 wt.% of MWCNTs; and (**b**) 0.3–1 wt.% of MWCNTs.

Figure 12a,b show the dynamic process of filler conductive network formation, which was observed by measuring the electrical conductivity as a function of time for the PP/PS/MWCNTs composite at 135 °C. It can be seen that, at a very low concentration of MWCNTs, the electrical conductivity was constant for around 11.5 min of treatment and then drastically increases by five orders of magnitudes (Figure 12a). The electrical conductivity of composites with 0.1 wt.% of MWCNTs is low at the start of the treatment due to the poor connection of nanoparticles inside the polymer matrix, but when the critical point of particle’s connections is achieved, they create an electric pathway and the electrical conductivity rapidly increases. Figure 12b shows the electrical conductivity as a function of time for the PP/PS/MWCNTs composite with 0.3–1 wt.% of MWCNTs. It can be seen that the electrical conductivity starts to increase from the first seconds of treatment and approaches a plateau after 5 min. In this case, the concentration of nanoparticles was sufficient for the creation of a conductive network from the start of the treatment.

Similar behavior was observed for the electrical conductivity of PP/MWCNTs as a function of time (as can be seen in Figures S4 and S5 in the Supplementary Materials). At very low concentrations of MWCNTs (0.1–0.3 wt.% of MWCNTs—below the percolation threshold), electrical conductivity did not change during the isothermal treatment, and its values were close to those of unfilled PP. However, at MWCNTs concentrations close to the PT and higher (0.5–1 wt.% of MWCNTs), electrical conductivity monotonically increased with time.

## 4. Discussion

There are several ways to reduce the PT of CNT-based thermoplastic composites prepared by melt mixing. One of these ways is to use immiscible polymer blends with a co-continuous morphology as a matrix. In this case, the reduction in PT can be achieved thanks to a double percolation effect, resulting from the co-continuous morphology of the used PB [6,9,24,25,26]. Furthermore, the reduction in PT can be achieved through both covalent and noncovalent modifications of either CNTs or matrices of CNTs-based thermoplastic composites with co-continuous morphology [7,25,28]. Some researchers suggested adding different nanoparticles to help trap CNTs at the interface [41,46]. Another way is to optimize the mixing parameters (time, mixing speed, …), as well as use varied post-mixing thermal treatments, as was discussed in the introduction [11,35,49,51]. Table 2 summarizes some literature studies in which the ultra-low PT of PB/CNTs composites was achieved along with the employed modifications and treatments. The PT reached in the present work for PP/PS/MWCNTs composites was comparable or even lower than those reported in the literature. The electrical conductivity values, achieved in this study, for several PT concentrations as well as 1 wt.% of CNTs, were greater than those reported in other studies. These values were obtained primarily due to the induced crystallization of PP during the post-mixing thermal treatments. As a result, ultra-low PTs for PP/PS/CNTs composites have been achieved.

The results reported in this work also showed that the thermal treatments did not influence the blend morphology as is the case after an annealing treatment performed above the melting or softening temperature, during which the coarsening of the PB matrix morphology is happening [11,29,30,31,49]. 

**Figure 13:**
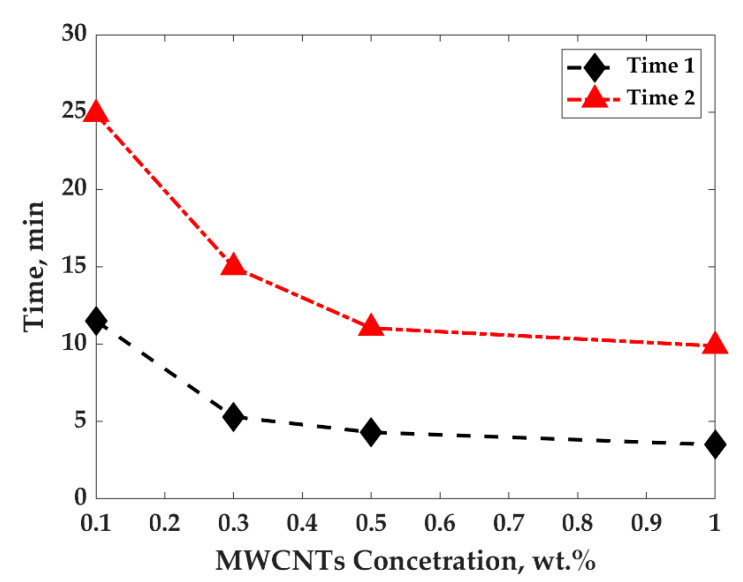
Time for complete crystallization of PP (Time 2) and time when electrical conductivity levels off (Time 1) as a function of MWCNTs concentration for PP/PS/MWCNTs composites.

The dynamic percolation threshold for the system studied in the present work was also investigated using electrical conductivity measurements. It was found that the electrical conductivity of PP/PS/MWCNTs composites with the lower concentration of 0.1 wt.% of MWCNTs (close to PT concentration) drastically increased after 11.5 min of thermal treatment at 135 °C, as can be observed in Figure 12a. For the samples with a larger MWCNTs concentration, the electrical conductivity monotonically increased with time, depending on the MWCNTs concentration. Figure 13 shows the time which corresponds to the complete crystallization of PP as a function of MWCNTs concentration, as well as the time which the electrical conductivity take to reach plateau values as a function of the MWCNTs concentration for PP/PS/MWCNTs composites. It can be seen that the electrical conductivity reached plateau values before the crystallization was complete. This is an indication, we believe, of an improvement of MWCNTs particle connections at the PP/PS interface. In this case, MWCNTs were pushed to PP crystals’ borders where they stopped by the PS phase which does not crystalize. Generally, the PS phase is more favorable for MWCNTs, but their diffusion into it is significantly impeded upon because the treatment temperature does not provide enough energy to promote MWCNTs diffusion. As a result, due to the accumulation of MWCNTs connections at the PP/PS interface and in the PP phase, the electrical conductivity increases.

## 5. Conclusions

PP/PS/MWCNTs blend composites with co-continuous morphology have been prepared by melt-mixing, using a twin-screw extruder through the dilution of a masterbatch of PP/MWCNTs with PP and PS. Due to the effect of double percolation, the PT of PP/PS/MWCNTs was reduced by over 50% compared to PP/MWCNTs composites. Furthermore, thermal annealing treatments, aimed at enhancing the effect of PP crystal growth on electrical conductivity and PT of PP/PS/MWCNTs composites have been done. It was shown that extremely low PTs of 0.06 wt.% and 0.08 wt.% MWCNTs were obtained after slow-cooling and isothermal treatments, respectively. These treatments promoted the selective localization of MWCNTs due to the PP crystal volume exclusion effect of MWCNTs. Moreover, microscopy observations (SEM) and the characteristic domain sizes calculation of PP/PS/MWCNTs co-continuous morphology confirmed that these treatments have not changed PB morphology in contrast to thermal annealing above the melting or softening temperature.

## Figures and Tables

**Table 1:** Percolation threshold and fitting values of experimental data according to Equation (1) for PP/PS/MWCNTs composites after each treatment.

Parameters	Fast-Cooling Treatment	Isothermal Treatment	Slow-Cooling Treatment
p_c_, wt.%	0.28	0.08	0.06
k, S/m	8.5 × 10^−4^	0.12	0.15
t	1.60	2.20	2.00
R^2^	0.97	0.89	0.99

**Table 2:** Ultra-low PT values of CNT-thermoplastic systems achieved by different modifications and treatments.

System	PT, wt.%	EC at PT, S/m	EC, S/m of PB with 1 wt.% of CNTs	Modification	Reference
PS/EVA/IL-CNTs (70/30 wt.%)	0.050	10^−9^	5.0 × 10^−1^	Modification of MWCNTs with noncovalent ionic liquid	[7]
PS/PBAT/IL-CNTs (50/50 wt.%)	0.050	10^−6^	5.0 × 10^−1^	Modification of MWCNTs with noncovalent ionic liquid	[28]
PS/PMMA/MWCNTs-COOH (40/60 wt.%)	0.017	10^−10^	5.0 × 10^−2^	MWCNTs functionalization with carboxyl groups	[25]
PLLA/EVA/GO0.3/CNTs (60/40 wt.%)	0.060	10^−9^	2.0 × 10^−5^	Trapping CNTs at the interface with the help of 0.3 wt.% of GO	[41]
PS/PVDF/Clay0.1/CNTs (40/60 wt.%)	0.060	10^−9^	5.0 × 10^−5^	Trapping CNTs at the interface with the help of 0.1 wt.% of organoclay	[46]
PLA/PCL/MWCNTs (50/50 wt.%)	0.025	10^−9^	2.0 × 10^−4^	Adjustment of melt-mixing time	[40]
PP/PMMA/MWCNT (30/70 wt.%)	0.090	10^−7^	2.0 × 10^−1^	2 h of annealing at 200 °C	[49]
PP/PS/MWCNTs (50/50 wt.%)	0.060	10^−6^	5.6 × 10^−1^	Slow-cooling treatment (takes 50 min)	Our work
PP/PS/MWCNTs (50/50 wt.%)	0.080	10^−5^	5.1 × 10^−1^	Isothermal treatment (takes 15 min)	Our work

## Data Availability

The data presented in this study are available in this article.

## Supplementary Materials

The following are available online at https://www.mdpi.com/article/10.3390/nano11061620/s1, Figure S1: Viscosity as a function of shear rate measured by capillary rheometer for pure PP and PS. Figure S2: Effect of treatments on electrical conductivity as a function of MWCNTs concentration for PP/MWCNTs composites. Figure S3: Image treatment analysis for distinguishing between two phases: (a) original SEM picture for PP/PS/MWCNTs composite with 0.3 wt.% of MWCNTs; (b) image phase separation; (c) final treated image which was used for the estimation of $L_{int}$—interface length between two phases, which in this case is the perimeter of the white phase (PS phase). Table S1: Percolation threshold and fitting values of experimental data according to Equation (6) for PP/MWCNTs composites after each treatment. Figure S4: Electrical conductivity as a function of time for PP/MWCNTs composite with 0–0.3 wt.% of MWCNTs measured every 10 s at 1 Hz of frequency and 135 °C. Figure S5: Electrical conductivity as a function of time for a PP/MWCNTs composite with 1 wt.% of MWCNTs measured every 10 s at 1 Hz of frequency and 135 °C.
