# Supplementary material for: Ultra-Low Percolation Threshold Induced by Thermal Treatments in Co-Continuous Blend-Based PP/PS/MWCNTs Nanocomposites
Source: Nanomaterials (Basel). 2021 Jun 21;11(6):1620. doi: 10.3390/nano11061620 (PMC8234504; doi:10.3390/nano11061620)
Supplement: Supplementary file 1 [file nanomaterials-11-01620-s001.zip › nanomaterials-1246145-supplementary.pdf]

Supplementary Material

Ultra-Low Percolation Threshold Induced by Thermal Treatments in Co-Continuous Blend-Based PP/PS/MWCNTs Nanocomposites

Daria Strugova, José Carlos Ferreira Junior, Éric David and Nicole R. Demarquette *

Mechanical Engineering Department, École de Technologie Supérieure, Montréal, Québec H3C 1K3, Canada; daria.strugova.1@ens.etsmtl.ca (D.S.); jose-carlos.ferreira-junior.1@ens.etsmtl.ca (J.C.F.J.); Eric.David@etsmtl.ca (É.D.)

* Correspondence: NicoleR.Demarquette@etsmtl.ca

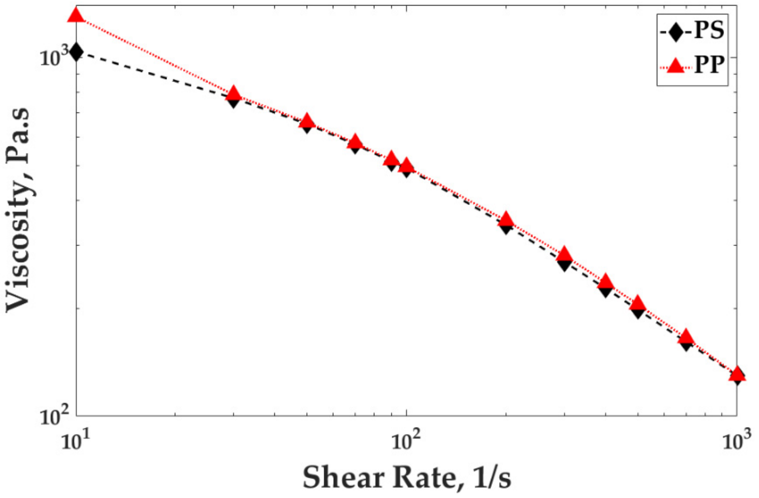

Figure S1. Viscosity as a function of shear rate measured by capillary rheometer for pure PP and PS.

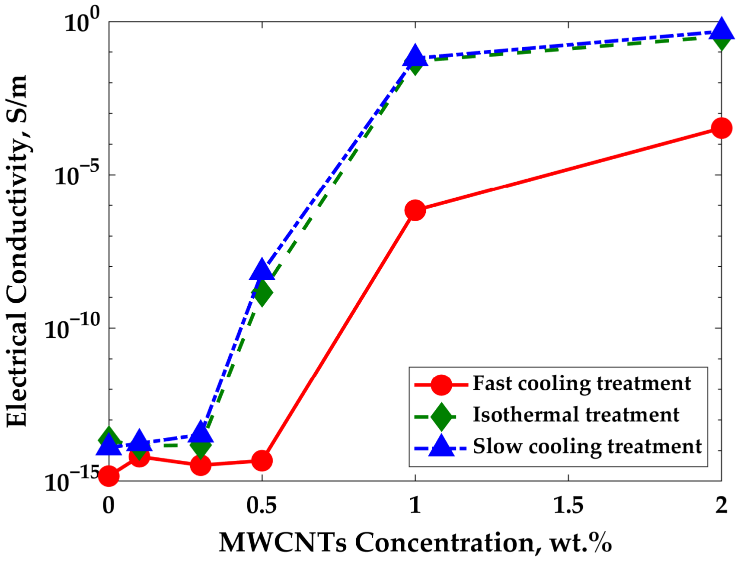

Figure S2. Effect of treatments on electrical conductivity as a function of MWCNTs concentration for PP/MWCNTs composites.

Table S1. Percolation threshold and fitting values of experimental data according to the equation 1 for PP/MWCNTs composites after each treatment.

Parameters	Fast cooling treatment	Isothermal treatment	Slow cooling treatment
**p_c, wt.%**	0.6	0.44	0.38
**k, S/m**	6.5×10^{-5}	0.15	0.17
**t**	4.9	1.8	2.1
**R^2**	1	0.95	0.93

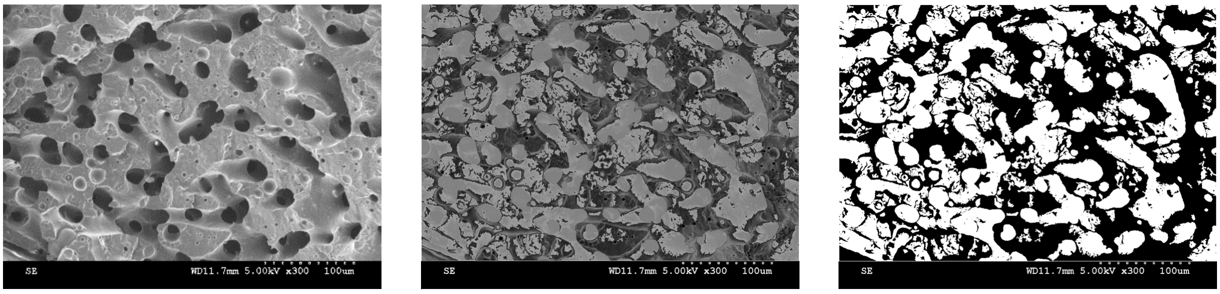

(**a**) (**b**) (**c**)

Figure S3. Image treatment analysis for distinguishing between two phases: (**a**) original SEM picture for PP/PS/MWCNTs composite with 0.3 wt.% of MWCNTs; (**b**) image phase separation; (**c**) final treated image which was used for the estimation of L_{int} - interface length between two phases, which in this case is the perimeter of white phase.

Table S2. Melting and crystallization temperatures, onset and end of crystallization and degree of crystallinity for PP/MWCNTs and PP/PS/MWCNTs nanocomposites.

Material	CNTs amount, wt.%	Peak of Tm, °C	Peak of Tc, °C	Onset Tc, °C	End Tc, °C	Xc, %
PP/CNTs	0	166 ± 0.2	112 ± 0.1	117 ± 0.2	107 ± 1.2	47 ± 0.09
	0.1	165 ± 0.2	121 ± 0.1	126 ± 0.2	118 ± 0.1	48 ± 0.08
	0.3	166 ± 0.3	124 ± 0.2	128 ± 0.1	120 ± 0.2	47 ± 0.1
	0.5	167 ± 0.9	126 ± 0.2	130 ± 0.1	121 ± 1.2	49 ± 1.8
PP/PS/CNTs	0	166 ± 0.5	117 ± 0.1	122 ± 0.1	113 ± 0.1	52 ± 1.4
	0.1	165 ± 1.3	119 ± 0.2	123 ± 0.1	114 ± 0.4	51 ± 0.2
	0.3	167 ± 0.3	121 ± 0.2	126 ± 0.5	116 ± 0.1	48 ± 0.8
	0.5	168 ± 0.2	122 ± 0.2	127 ± 0.4	117 ± 0.1	52 ± 0.6

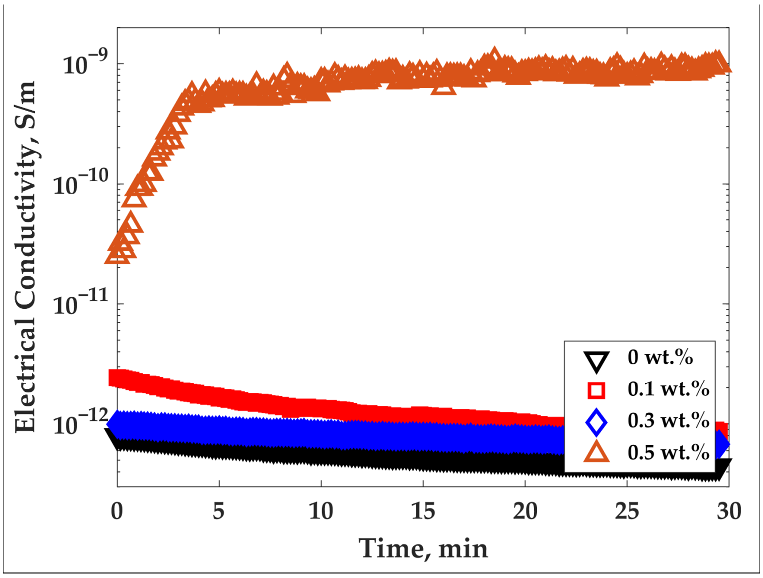

Figure S4. Electrical conductivity as a function of time for PP/MWCNTs composite with 0-0.3 wt.% of MWCNTs measured every 10 s at 1 Hz of frequency and 135 °C.

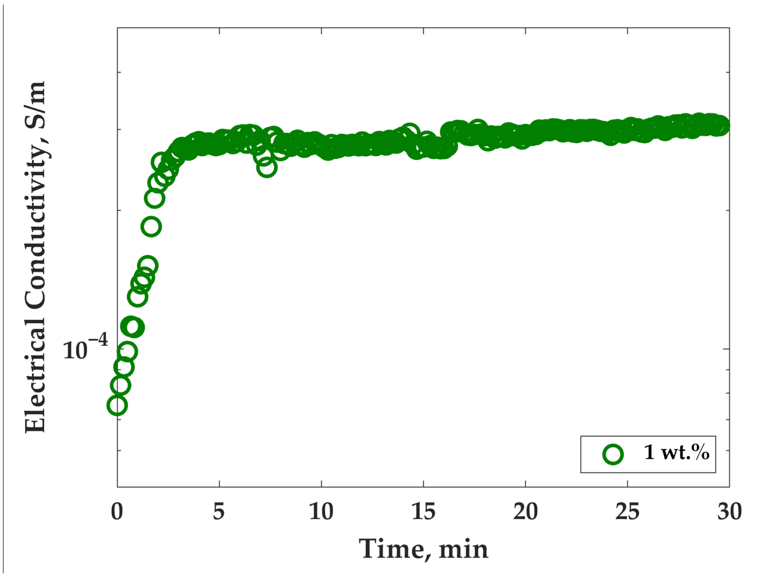

Figure S5. Electrical conductivity as a function of time for PP/MWCNTs composite with 1 wt.% of MWCNTs measured every 10 s at 1 Hz of frequency and 135 °C.
